# Congenital Complete Atrioventricular Block With Anti–Sjögren Syndrome–Related Antigen A Antibody–Impaired Myocardium: A Case Report

**DOI:** 10.1016/j.cjcpc.2026.01.004

**Published:** 2026-02-17

**Authors:** Hitomi Ono, Yuichiro Takahashi, Ryuichi Shimaoka, Masako Matsui, Kazuhiko Asai, Shigenori Iwagaki, Masaki Katayama

**Affiliations:** a Department of Fetal-maternal Medicine and Obstetrics, Gifu Prefectural General Medical Center, Gifu, Japan; b Department of Diagnostic Pathology, Gifu Prefectural General Medical Center, Gifu, Japan

**Keywords:** complete atrioventricular block, hydrops fetalis, impulse conducting system

**Congenital complete atrioventricular block (CAVB) occurs in 1%-2% of pregnancies positive for Sjögren syndrome–related antigen A (Ro/SSA) and B (La/SSB) antibodies.****^1^** **We report a case of CAVB with extensive impairment of the impulse-conducting myocardium in an infant born at 21 weeks 2 days to an anti-SSA–positive mother with Sjögren syndrome. Pathologic examination revealed widespread damage to the myocardium. Immunohistochemistry (IHC) showed strong anti-Ro52 staining in the affected myocardium with colocalization of maternal IgG, which also cross-reacted with fetal anti-Ro52. This report highlights the immunoreactive basis of myocardial degeneration along the impulse-conducting system in CAVB.**

## Case Presentation

A 28-year-old primigravida visited our hospital for second-trimester screening at 20 + 0 weeks of gestation. We identified a fetus with severe whole-body skin edema and pleural effusion. Fetal echocardiography indicated bradycardia and high echogenicity of the right ventricle. M-mode recordings demonstrated a slow ventricular rate and complete dissociation between the atrial and ventricular contractions (atrial rate, 141 bpm; ventricular rate, 88 bpm). The patient was diagnosed with CAVB. No structural heart defects or congenital anomalies were detected. Immunologic testing revealed strong positivity for serum SSA (Ro) antibodies (>240-fold), whereas SSB (La) antibody and indirect Coombs test results were negative. Infections, including toxoplasmosis, syphilis, Zika virus, varicella-zoster virus, rubella, cytomegalovirus, and herpes simplex virus, were excluded. We anticipated a poor fetal prognosis, and after counseling, the patient and her husband opted for pregnancy termination. A female infant weighing 454 g was born at 21 + 2 weeks of gestation. After delivery, the patient was diagnosed with Sjögren syndrome.

**Figure 1:**
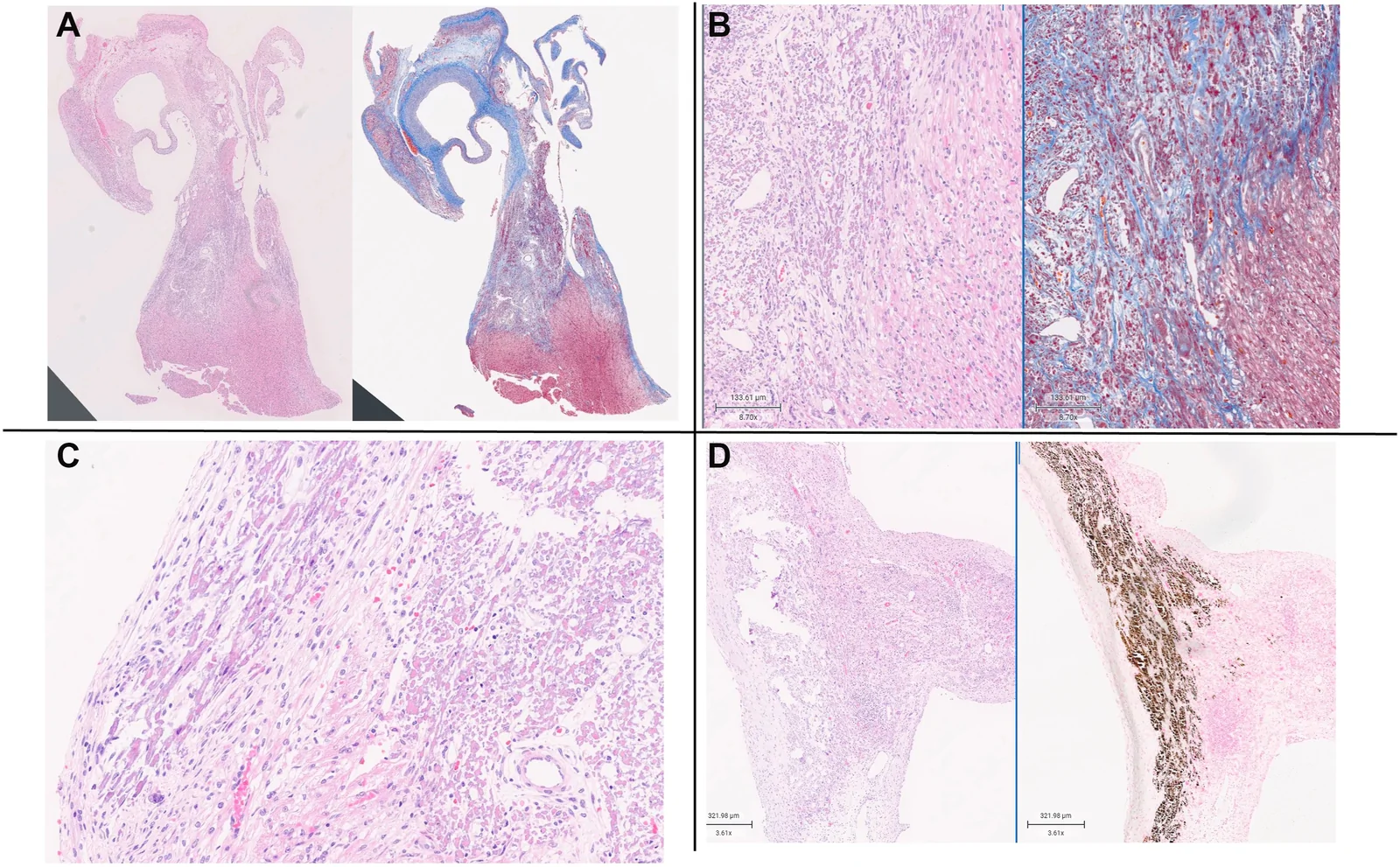
(**A**) Atrioventricular node. Left, hematoxylin-eosin (H&E) staining; right, Masson's trichrome (MT) staining. (**B**) Enlarged view of (**A**). Myocardial fibers were ruptured, with inflammatory cell infiltration (H&E staining). This indicated replacement by collagen fibers in the MT staining. (**C**) Right branch of the His bundle (H&E staining). The myocardial fibers were ruptured. This was more pronounced in the right branch than in the left branch. (**D**) Atrial septum. Left H&E staining; myocardial fibers were ruptured, with inflammatory cell infiltration. The right panel presents von Kossa staining, which revealed calcification.

**Figure 2:**
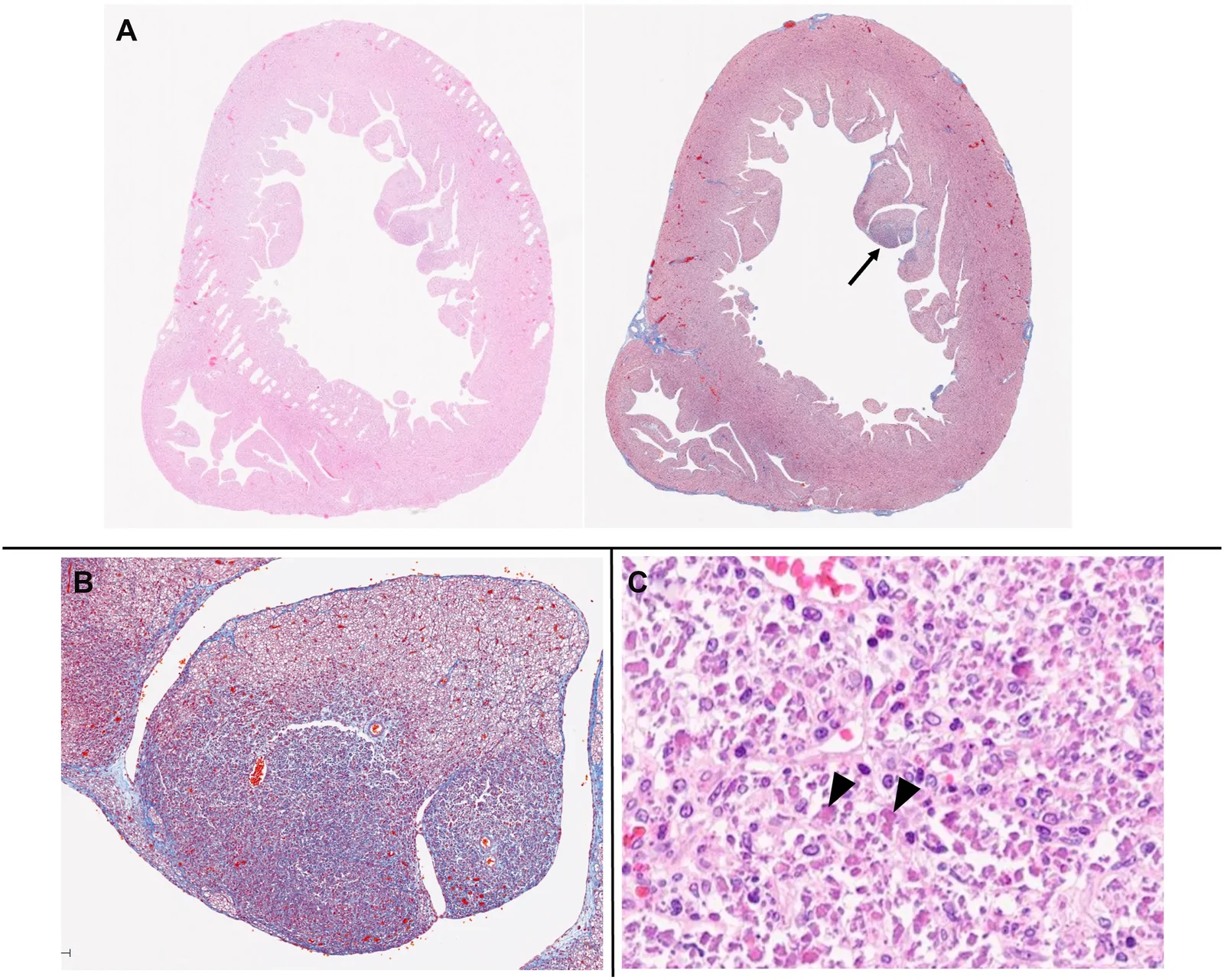
(**A**) Right and left ventricles. Left, hematoxylin-eosin staining; right, Masson's trichrome staining. Infiltration of inflammatory cells and replacement by collagen fibers in parts of the endocardium was remarkable, particularly in the right ventricle. (**B**) Enlarged view of (**A**) (arrow), and collagen fibers replaced them. (**C**) Enlarged view of (**B**), demonstrating calcium salt deposits.

We performed a cardiac autopsy on the baby. The heart was dilated but structurally normal. The myocardial samples of the coronal section of both ventricles and the atrioventricular and sinus nodal areas were processed for light microscopy. Cardiac histology indicated extensive damage to myocytes along the conduction system of the fetal heart. In the atrioventricular node, coagulation necrosis of myocytes, lymphocyte infiltration, and replacement fibrosis were observed (Fig. 1, A and B). Myocardial degeneration was observed in both bundles of His, being more remarkable in the right branch (Fig. 1C). In addition, replacement fibrosis and prominent calcification were observed in the atrial septum (Fig. 1D; von Kossa staining). Coronal sections of the ventricles exhibited focal endocardial degeneration and fibrosis, similar to that in the atrioventricular node (Fig. 2). No abnormalities were observed in the sinus nodes.

We performed IHC staining of the same tissue section to assess the association between myocardial degeneration and maternal SSA antigen. Impaired myocytes surrounding the fibrotic area tested strongly positive for anti-Ro52 antibody (Supplemental Fig. S1). Human IgG antibodies exhibited diffuse deposition in the replacement fibrosis area (Supplemental Fig. S2). Furthermore, to clearly assess the cross-activity of maternal IgG, we performed IHC staining using maternal serum as the primary antibody source and horseradish peroxidase-labeled anti-human IgG as the secondary antibody. The myocytes were positive for maternal sera, whereas the control condition (secondary antibody only) yielded negative results (Supplemental Fig. S3).

## Discussion

Here, we present a case of CAVB with an extensively impaired myocardium along an impulse-conducting system. We identified only 14 reports on pathologic findings of CAVB associated with maternal anti-SSA/SSB antibodies or systemic lupus erythematosus. With a median diagnosis at 20 (17-36) weeks, 2 intrauterine fetal deaths, 1 termination of pregnancy, 6 neonatal deaths, 3 infant deaths, and 2 cases with unknown outcomes were reported. The most common pathologic findings were abnormalities of the atrioventricular node, such as fibrosis and calcification. In addition, 5 studies reported endocardial fibroelastosis. Few reports mentioned findings of bundles of His, such as rupture, partial aplasia, fibrosis, and calcification.^2^, ^3^, ^4^ To the best of our knowledge, this is the first report on fetal CAVB with extensive pathologic impairment of the myocardium along the impulse-conducting system. In the present case, myocardial degeneration was more pronounced in the right ventricle. Severe right ventricular dysfunction may cause hydrops fetalis, which explains its high echogenicity.

IHC analysis revealed 3 novel outcomes. First, impaired myocytes along the impulse-conducting system of the fetal heart were strongly positive for the anti-Ro52 antibody, whereas normal myocytes were negative. Ro52 is highly expressed in impaired cardiomyocytes. Previous reports demonstrated that anti-Ro52 antibodies are involved in the dysregulation of calcium channels that are highly expressed in the atrioventricular and sinoatrial nodes. Consequently, cardiomyocyte apoptosis is induced, and Ro52, initially localized in the nucleus, is translocated to the cell membrane surface in apoptotic cells.^5^, ^6^, ^7^ Our IHC staining results supported these previous findings. Second, IgG deposition was observed in the regions corresponding to the damaged areas. This indicates that transplacentally transferred maternal autoantibodies accumulate in these regions. Finally, the autoantibodies present in the maternal serum cross-reacted with fetal cardiomyocytes, confirming that maternal autoantibodies trigger an autoimmune response in fetal cardiomyocytes. This is likely to be the first report to demonstrate the fetal heart's immunoreaction with CAVB, particularly the cross-reactivity between maternal IgG and anti-Ro52.

## Conclusions

We pathologically demonstrated extensive impairment of the myocardium along the impulse-conducting system in a fetus with CAVB and hydrops fetalis. IHC revealed cross-reactivity between maternal IgG and anti-Ro52 in the damaged fetal myocardium. Prominent ventricular hyperintense echograms may signify severe myocardial degeneration and provide a clue for the prediction of fetal prognosis.

**Novel Teaching Points**

• Maternal anti–Sjögren syndrome–related antigen A and B antibodies may induce fetal congenital complete atrioventricular block and extensive impairment of the myocardium along the impulse-conducting system.
• High echogenicity of the right ventricle suggests severe myocardial damage and dysfunction and may cause hydrops fetalis.
• Immunohistochemistry staining revealed a cross-reaction between maternal IgG and anti-Ro52 in the impaired fetal heart.

## Data Availability

All data generated or analyzed during this study are included in this article. Further inquiries can be directed to the corresponding author.

## Ethics Statement

According to local guidelines, ethical approval was not required for this study.

## Patient Consent

The authors confirm that patient consent forms have been obtained for this article.

## Funding Sources

The authors have no funding sources to declare.

## Disclosures

The authors have no conflicts of interest to disclose.
