# Supplementary material for: Congenital Complete Atrioventricular Block With Anti–Sjögren Syndrome–Related Antigen A Antibody–Impaired Myocardium: A Case Report
Source: CJC Pediatr Congenit Heart Dis. 2026 Feb 17;5(4):242–5. doi: 10.1016/j.cjcpc.2026.01.004 (PMC13523816; doi:10.1016/j.cjcpc.2026.01.004)
Supplement: Supplementary file 1 [file mmc1.pdf]

Supplemental Figure S1

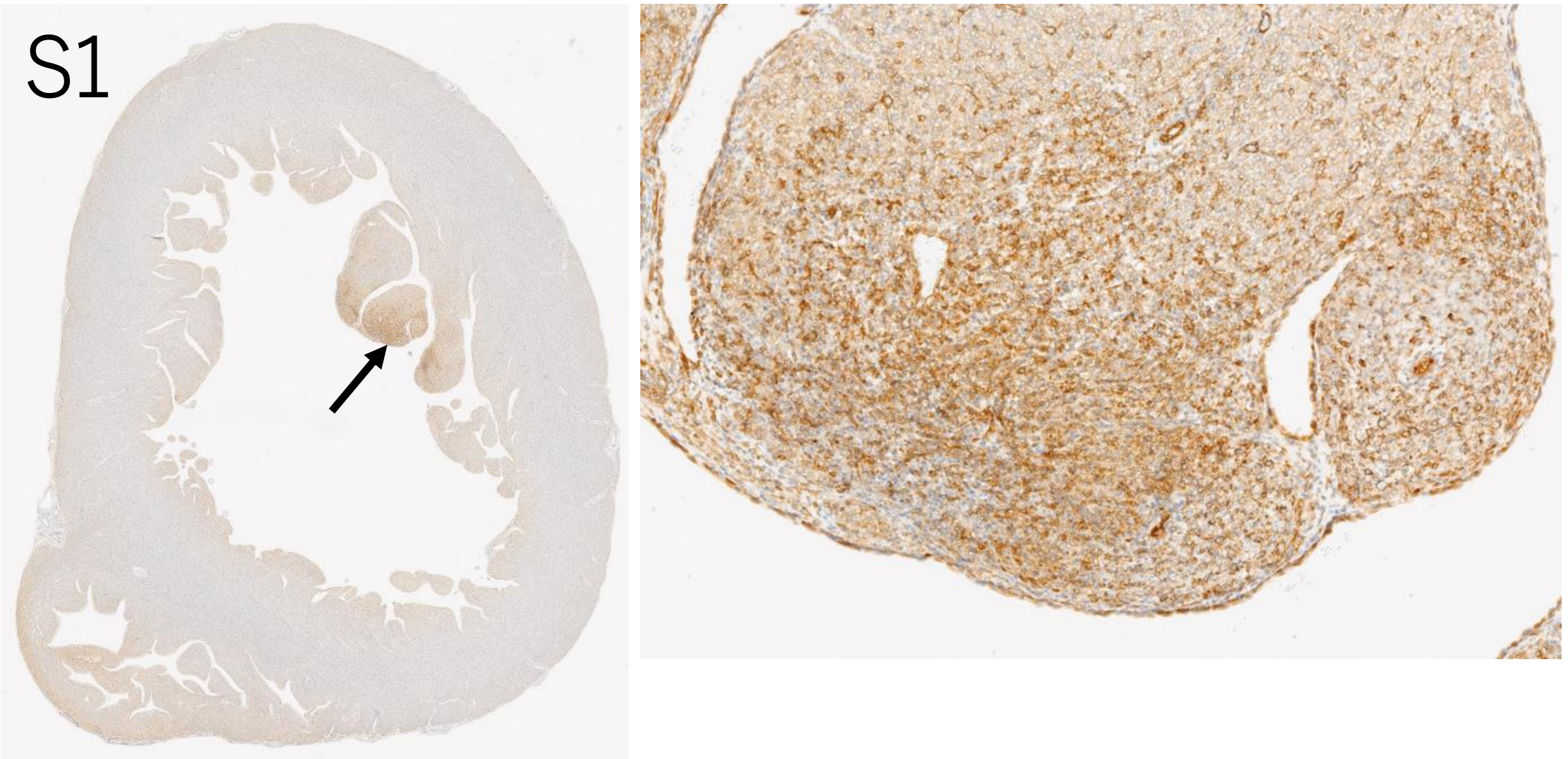


Supplemental Figure S2

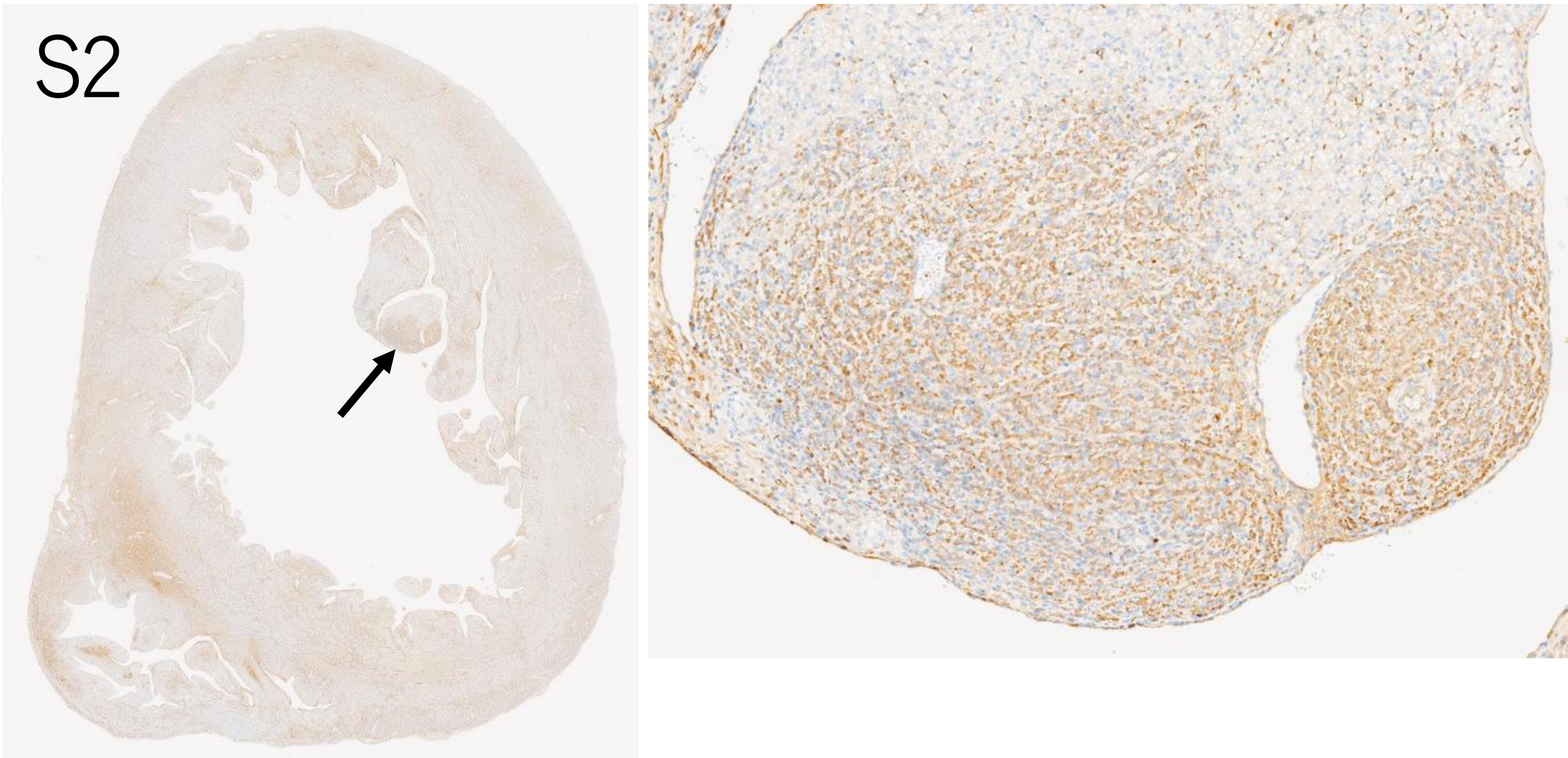


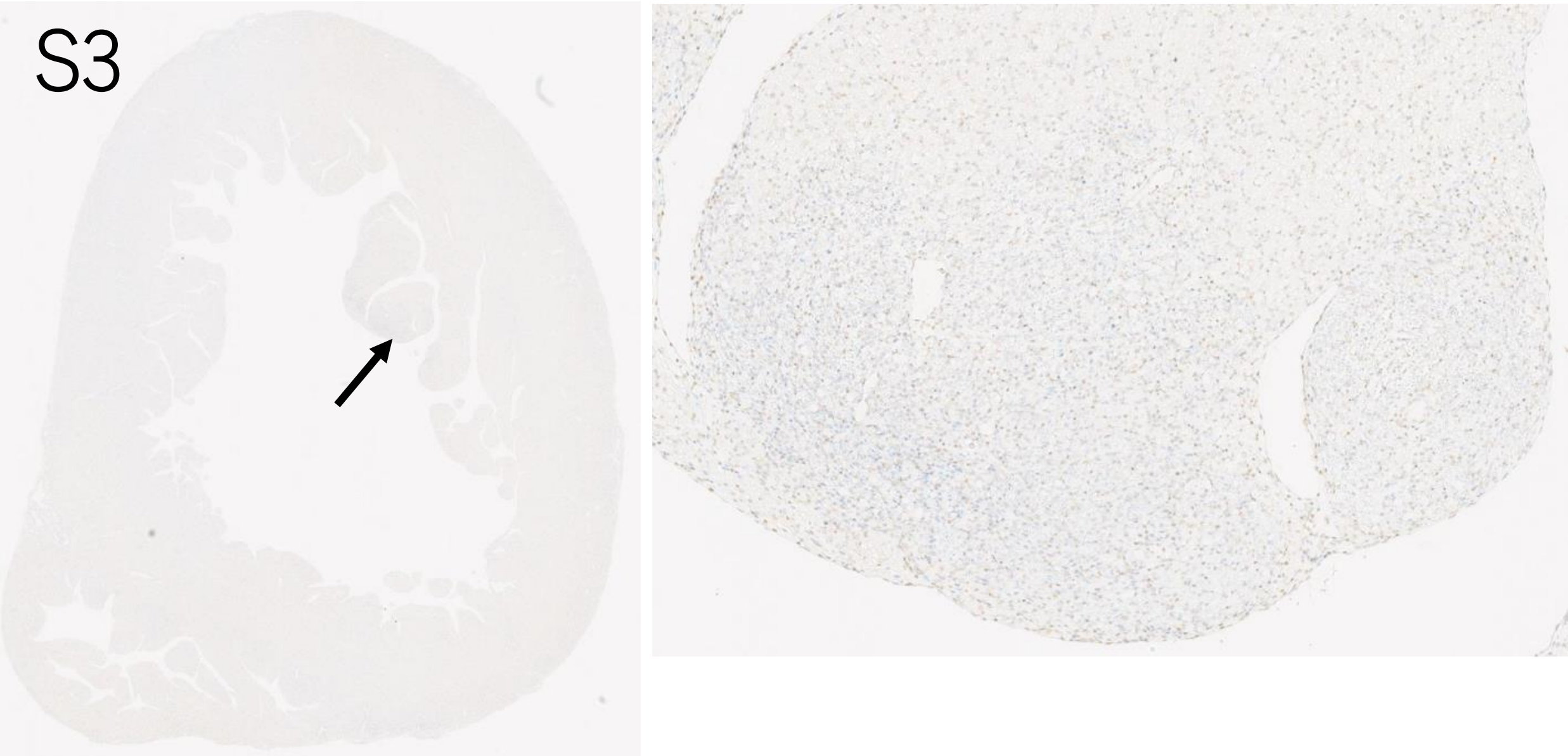


Supplemental Figure S3

Immunostaining images

(S1) First examination using an anti-Ro52 antibody. Impaired sections along the impulse-conducting system of the fetal heart were positively stained.

(S2) Second examination using rabbit anti-IgG antibody. The same sections also exhibited positive immunoreactivity.

(S3) Third examination using the patient's serum and rabbit anti-IgG antibody. The immunoreaction was positive and stronger in the tissue after adding the patient's serum.

IgG, immunoglobulin G
